# Impairment of the Organization of Locomotor and Exploratory Behaviors in Bile Duct-Ligated Rats

**DOI:** 10.1371/journal.pone.0036322

**Published:** 2012-05-07

**Authors:** Renata Leke, Diogo L. de Oliveira, Ben Hur M. Mussulini, Mery S. Pereira, Vanessa Kazlauckas, Guilherme Mazzini, Carolina R. Hartmann, Themis R. Silveira, Mette Simonsen, Lasse K. Bak, Helle S. Waagepetersen, Susanne Keiding, Arne Schousboe, Luis V. Portela

**Affiliations:** 1 Department of Biochemistry, ICBS, Federal University of Rio Grande do Sul, Porto Alegre, Rio Grande do Sol, Brazil; 2 Department of Pathology, Hospital de Clínicas de Porto Alegre, Porto Alegre, Rio Grande do Sol, Brazil; 3 Experimental Hepatology and Gastroenterology Laboratory, Research Center of Hospital de Clínicas de Porto Alegre, Post-Graduation in Child and Adolescents Health, Federal University of Rio Grande do Sul, Porto Alegre, Rio Grande do Sol, Brazil; 4 Positron Emission Tomography Centre, Aarhus University Hospital, Aarhus, Denmark; 5 Department of Medicine V, Hepatology and Gastroenterology, Aarhus University Hospital, Aarhus, Denmark; 6 Department of Drug Design and Pharmacology, Faculty of Health and Medical Sciences, University of Copenhagen, Copenhagen, Denmark; Radboud University, The Netherlands

## Abstract

Hepatic encephalopathy (HE) arises from acute or chronic liver diseases and leads to several problems, including motor impairment. Animal models of chronic liver disease have extensively investigated the mechanisms of this disease. Impairment of locomotor activity has been described in different rat models. However, these studies are controversial and the majority has primarily analyzed activity parameters. Therefore, the aim of the present study was to evaluate locomotor and exploratory behavior in bile duct-ligated (BDL) rats to explore the spatial and temporal structure of behavior. Adult female Wistar rats underwent common bile duct ligation (BDL rats) or the manipulation of common bile duct without ligation (control rats). Six weeks after surgery, control and BDL rats underwent open-field, plus-maze and foot-fault behavioral tasks. The BDL rats developed chronic liver failure and exhibited a decrease in total distance traveled, increased total immobility time, smaller number of rearings, longer periods in the home base area and decreased percentage of time in the center zone of the arena, when compared to the control rats. Moreover, the performance of the BDL rats was not different from the control rats for the elevated plus-maze and foot-fault tasks. Therefore, the BDL rats demonstrated disturbed spontaneous locomotor and exploratory activities as a consequence of altered spatio-temporal organization of behavior.

## Introduction

Hepatic encephalopathy (HE) is a neuropsychiatric complication that arises from acute or chronic liver diseases [1], [2]. According to the etiology of liver injury, HE is classified as follows: type A, derived from acute liver failure; type B, associated with hepatic portal-systemic vascular shunting in patients without liver disease; and type C, associated with liver cirrhosis and portal-systemic vascular shunting due to portal hypertension [3]. The clinical manifestations of HE range from sleep disturbances and slight attention deficits to somnolence-sopor and coma [4]. Moreover, patients with HE experience altered motor function, such as hypokinesia, bradykinesia, ataxia and asterixis [4], [5]. These psychomotor manifestations and the cognitive and emotional behavior dysfunctions impair the quality of life of HE patients [6], [7].

Various models of chronic liver disease in rats leading to HE type B and C have been used to study changes in biochemical and psychomotor activities, such as bile duct-ligated (BDL) rats [8]–[11], porto-caval anastomosis (PCA) [12]–[14], portal vein-ligated (PVL) [15] and thioacetamide-induced liver damage [16]. Most studies report a decreased spontaneous locomotor activity in these animals [9], [12], [14], but some results are controversial, describing no differences in locomotor activity between PCA and control rats [17], [18] and even increased locomotor activity in PCA rats compared to control rats [19]. This discrepancy might be due to the diversity of the animal models employed, the protocols and the analysis of the locomotor and exploratory behaviors. The majority of the psychomotor studies in rats analyzed the total distance traveled or the number of crossings in the arena as well as the total number of rearings, which primarily describe the quantity of activity. However, these studies did not explore the sequential (spatio-temporal) structure of behavior [20].

A frequently used animal model is the BDL rat, which according to the members of the ISHEN (International Society for Hepatic Encephalopathy and Nitrogen Metabolism) commission on experimental models of HE, reflects HE associated with cirrhosis and portal hypertension [21], [22]. It is described that animals that are subjected to this surgical model of type C HE develops liver failure, jaundice, portal hypertension, bacterial translocation, immune system dysfunction, hyperammonemia and low-grade encephalopathy [21]. Using this animal model we have obtained evidence that the biosynthetic machinery for transmitter GABA is disturbed [23], which may be of interest since GABAergic neurotransmission has been related to aberrations in the behavioral parameters that are normally associated with HE [12], [24].

**Table 1:** Biochemical parameters.

*Parameters*	*Control*	*BDL*	*t*	*df*	*p*
AST (U/L)	37.83±2.48	93.67±9.19^*^	7.721	16	<0.0001
Alkaline Phosphatases (U/L)	65.83±3.76	340.83±24.41^*^	15.66	16	<0.0001
Direct Bilirubin (mg/dL)	0.001±0.00	6.43±0.28^*^	32.96	16	<0.0001
Total Bilirubin (mg/dL)	0.06±0.01	10.54±0.32^*^	47.73	16	<0.0001
Albumin (g/dL)	4.45±0.09	2.08±0.16^*^	14.20	16	<0.0001

Biochemical parameters in serum of control (n = 12) and BDL rats (n = 6). Data are presented as means ± SEM. ^*^  =  *p*<0.05 by Student's t test. df =  degrees of freedom.

On this basis, the present study was undertaken to investigate the structure of locomotor and exploratory behaviors in BDL rats in an open-field task. BDL rats exhibited an altered spatio-temporal organization of locomotor and exploratory behavior in the open-field behavioral task.

## Methods

### Animals

Adult female Wistar rats (n = 25, weight 182±2.5 g, 60 days old at the initiation of the surgical procedure) were obtained from the *animalarium* of the Biochemistry Department (Federal University of Rio Grande do Sul). The rats were housed three animals per cage and maintained in a controlled environment (room temperature 21±1°C, standard light/dark cycle of 12 h – lights on at 07:00 am) with standard food and water *ad libitum*. The handling and care of the animals were conducted according to the guidelines of the Guide for the Care and Use of Laboratory Animals (NIH-USA). The Ethics Committee of the Federal University of Rio Grande do Sul approved all procedures.

**Figure 1:**
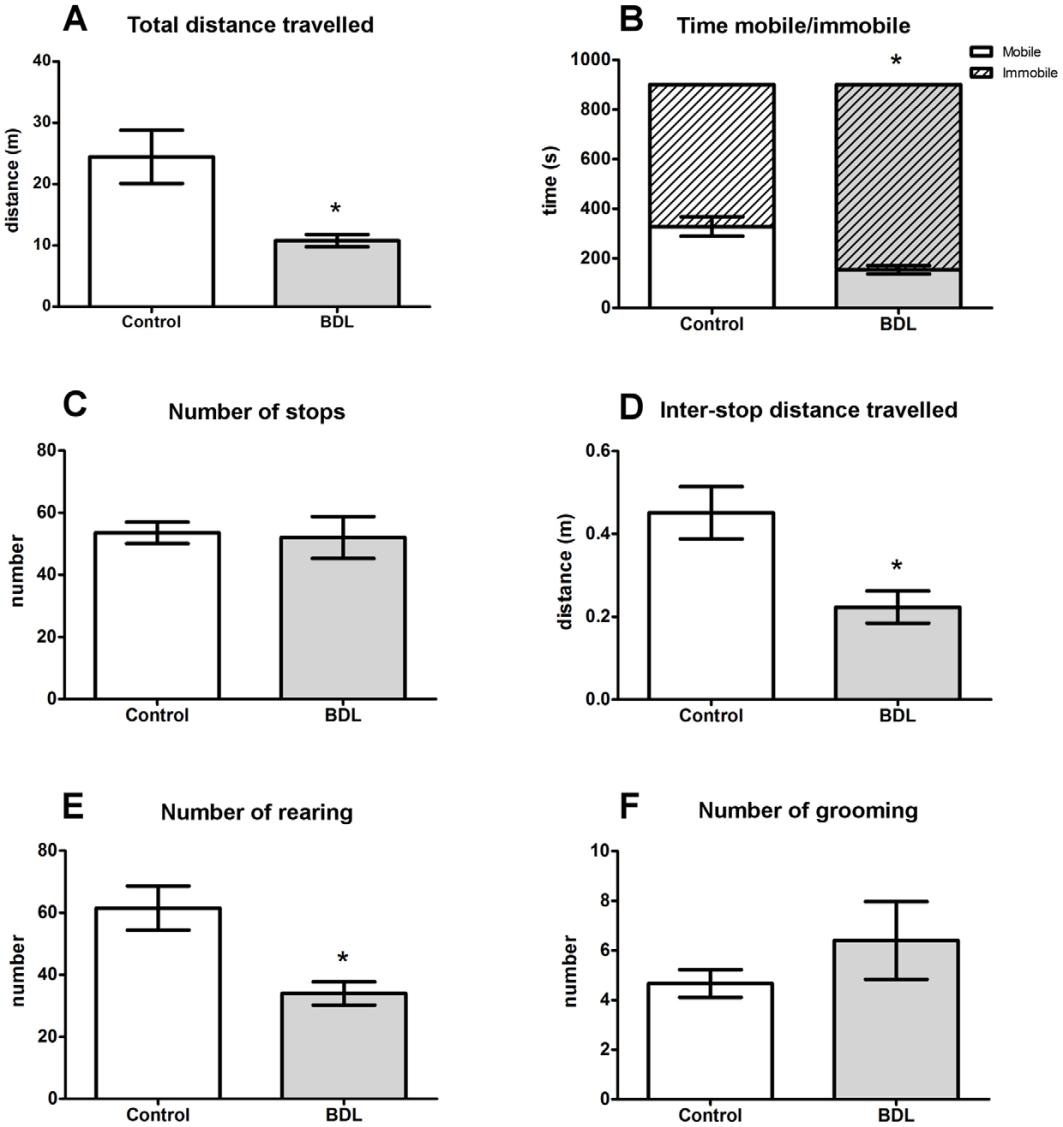
Locomotor and exploratory behavior. Control (n = 6) and BDL (n = 5) rats were evaluated for (A) total distance traveled, (B) time mobile/immobile, (C) number of stops, (D) inter-stops distance traveled, (E) number of rearings and (F) number of grooming. Data are presented as means ± SEM. *  =  *p*<0.05 by Student's t test.

**Figure 2:**
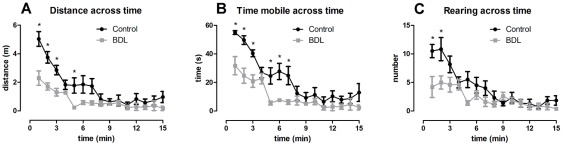
Locomotor and exploratory behavior across time. Control (n = 6) and BDL (n = 5) rats were evaluated for (A) distance traveled across time, (B) time mobile across time, (C) number of rearings across time. Data are presented as means ± SEM. *  =  *p*<0.05 by two-way ANOVA followed by Bonferroni post-hoc test.

**Figure 3:**
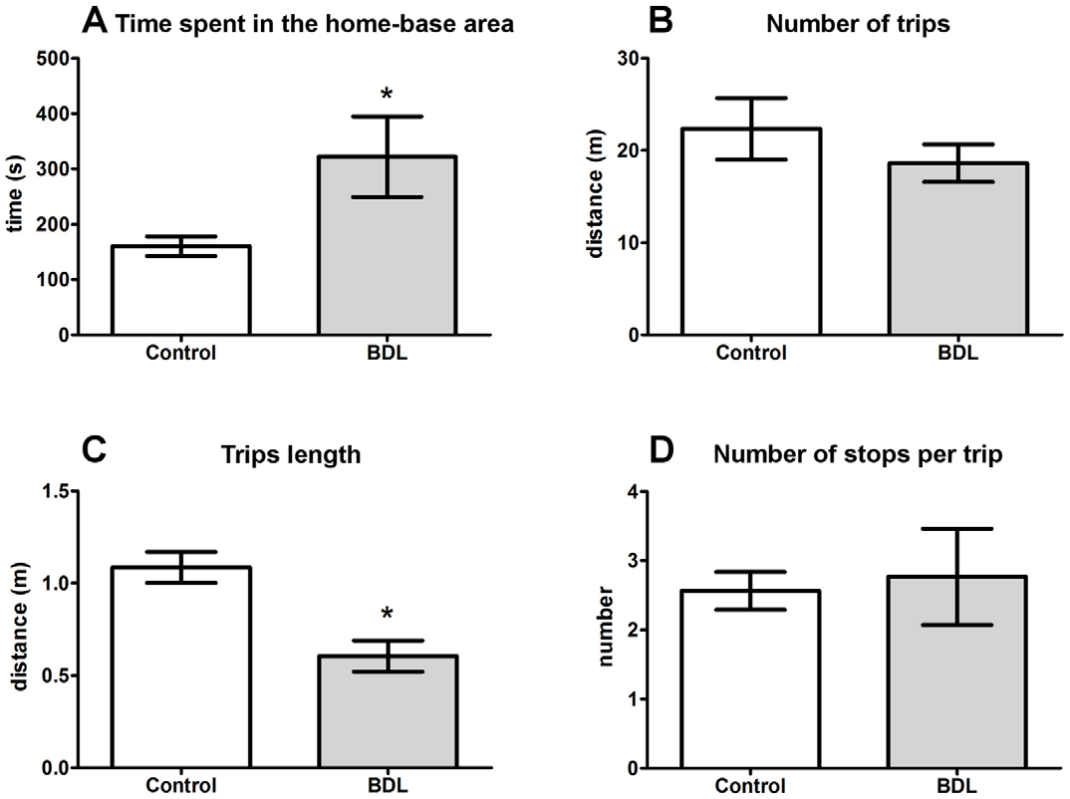
Temporal organization of behavior. Control (n = 6) and BDL (n = 5) rats were evaluated for (A) time spent in the home base area, (B) number of trips, (C) trips length and, (D) number of stops per trip. Data are presented as means ± SEM. *  =  *p*<0.05 by Student's t test.

**Figure 4:**
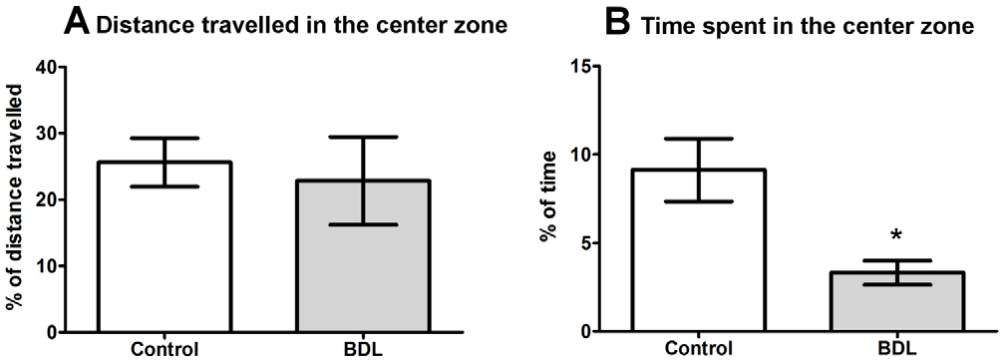
Spatial distribution of behavior. Control (n = 6) and BDL (n = 5) rats were evaluated for (A) percentage of total distance traveled in the center zone and (B) percentage of time spent in the center zone. Data are presented as means ± SEM. *  =  *p*<0.05 by Student's t test.

**Table 2:** Results of the plus-maze and foot-fault tasks.

Plus-maze					
*Parameter*	*Control*	*BDL*	*t*	*Df*	*p*
Number of entries (%)	55.84±5.60	49.75±3.06	1.117	9	0.2931
Time spent in the arm (%)	67.96±8.39	48.11±6.38	1.720	9	0.1196

Foot-fault					
*Parameter*	*Control*	*BDL*	*t*	*Df*	*p*
Number of faults	11.00±1.36	11.00±2.34	0.000	11	1.000

Control (N = 6) and BDL (N = 5) in the plus-maze and control (N = 8) and BDL (N = 5) in the foot-fault tasks. Data are presented as means ± SEM. Statistical analyses were performed using Student's t-test. df =  degrees of freedom.

**Figure 5:**
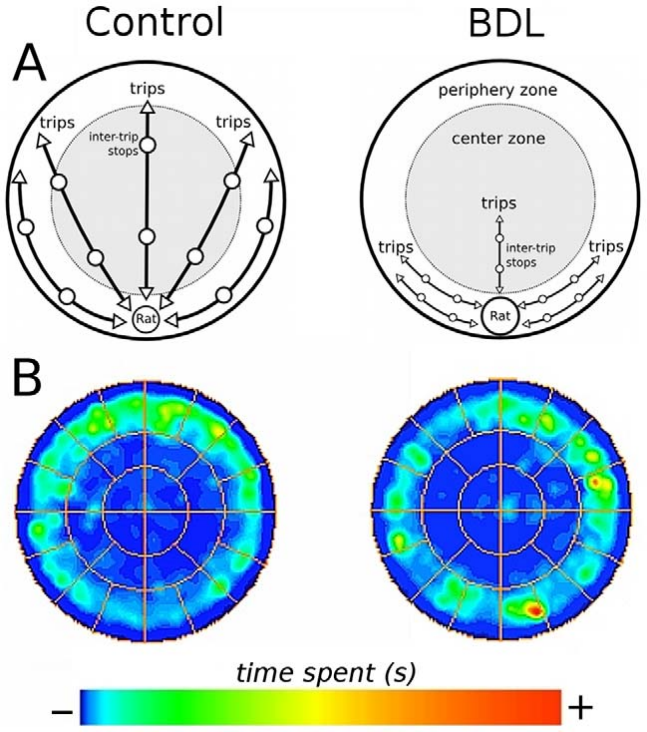
Schematic representation of behavior of control and BDL rats in the open-field task. (A) Representation of the spatio-temporal organization of locomotor and exploratory behavior in the control and BDL rats in the open-field task. (B) Occupancy plot of the open-field is presented as time (s) spent in the arena in the control and BDL groups. Control and BDL rats exhibited no differences in number of trips, number of stops and number of stops per trip during exploration of the arena; however, the length of the trips was shorter for BDL rats (A). BDL rats exhibited a decrease in the percentage of time spent in the center of the area and an increase in the time spent in the home base area (A and B).

### Bile duct ligation

The animals were randomly distributed into two groups: bile duct-ligated (BDL group, n = 10) and sham-operated (control group, n = 15) rats. The bile duct ligation procedure was conducted as described previously [22], [25]. The rats were anaesthetized with ketamine (90 mg/kg) and xylazine (12 mg/kg) i.p. and placed in the supine position on an operating table. A thermal controlled mattress (37°C) was used to assure a constant body temperature during the surgical procedure. A middle abdominal incision was performed and the hepatic ligament was exposed. The common bile duct was ligated with two 4-0 non absorbent surgical sutures. The first suture was placed below the junction of the biliary hepatic ducts and the second suture was placed above the entrance of the pancreatic ducts. The common bile duct was resected between the two ligatures. The abdominal incision was closed with 4-0 non absorbent suture in two layers. The rats were placed on another thermal mattress until consciousness was regained. The rats were then returned to their home-cages. The control rats consisted of sham-operated rats, i.e. rats that had the hepatic ligament exposed and manipulated, but the common bile duct was not ligated. All animals were maintained in the animal colony room for 6 weeks following surgery.

### Sample collection

Rats were anesthetized as described previously and blood was withdrawn by cardiac puncture. The animals were decapitated and the liver and spleen were removed and stored in a 10% formaldehyde solution (10% formaldehyde in 0.1 mM phosphate buffer containing 21.6 mM Na2HPO4 and 81 mM NaH2PO4, pH 7.4). All organs were stored at 4°C until histological analysis. Blood samples were centrifuged for 5 minutes at 5000×g and 20°C and serum samples were stored at −20°C until analysis.

### Histopathological examination

The livers and spleens of control and BDL rats were weighted and examined macroscopically. Subsequently, liver and spleen tissues were sampled for histological examination using Haematoxylin & Eosin (H&E) and Picrosirius (liver tissue) staining.

### Biochemical parameters

Serum samples from the control and BDL groups were analyzed for aspartate aminotransferase (AST), alkaline phosphatases, bilirubin (total and direct) and albumin using an auto-analyzer (Cobas Integra 400, Roche Diagnostics Corporation®, USA).

### Behavioral studies

The BDL and control rats were divided into two groups, 7 control and 5 BDL rats in the first group and 8 control and 5 BDL rats in the second group. The first group underwent open-field and plus-maze tasks, and the second group underwent foot-fault task. All experiments were conducted between the sixth and seventh week after surgical procedures. During the time between surgery and behavior tasks the rats were weekly manipulated to monitor weight gain and to avoid stress. Before each behavior task the rats were placed in the testing room (room size of 2 x 2.3 m, total area of 4.6 m^2^) for one hour to allow habituation with the environment and researchers. Inter-task intervals were 2 days and all behavioral tasks were performed between 9:00–16:00 h. All behavioral parameters were recorded and analyzed using the appropriate video tracking system (Any-maze®, Stoelting CO, USA).

### Open-field

Individual animals were placed in a 60-cm diameter circular black arena with 50-cm walls facing the wall. The animals were free to explore the arena for 15 min. The area was divided in two main sections, the center (0.1534 m^2^) and periphery (0.1293 m^2^) sections. Two halogen lamps (100 lx) pointed towards the room walls illuminated the room. After each trial, the apparatus was cleaned with 30% ethanol solution. The following parameters were quantified according to Eilam [20]: (1) activity (total distance traveled, time mobile/immobile, number of stops, inter stop distances, total numbers of rearing and grooming); (2) temporal organization of behavior (number of trips, trip lengths and the ratio between stops per trip); and (3) spatial distribution of activity (distance traveled within the center of the arena-calculated as a percentage of the total distance traveled, and time spent locomoting in the center of the arena-calculated as a percentage of the total locomotion time).

### Elevated plus-maze

Anxiety behavior was assessed in an elevated plus-maze according to Walf and Frye [26]. This task relies on the rodent's proclivity to dark enclosed spaces and an unconditioned fear of heights and open spaces. The apparatus consisted of four 50-cm long and 10-cm wide arms with two 40-cm high walls on two opposite arms. The apparatus was 50-cm above the floor and situated in the center of a red-lit room (60 lx). Red light was employed instead white light since it was demonstrated that the white light increases the open space avoidance [27]. The rats were placed individually at the center of the apparatus facing the open arm and they were free to explore it for 5 min. After each trial, the apparatus was cleaned with 30% ethanol solution. The following parameters were quantified: (1) percentage of the number of entries in the closed arms by the total number of entries into the closed and open arms, and (2) percentage of time spent in the closed arms by the total time spent in both the closed and open arms.

### Foot-fault

The rats were placed on a grid, and the number of failed attempts to accurately grasp the rungs was analyzed to examine coordinated fore- and hind-limb placement during spontaneous exploration. The apparatus was an elevated 80 cm×60 cm wire grid platform with 3-cm^2^ holes that was placed 76.5-cm above the floor. The animals freely explored the platform for 3 min. A foot-fault was scored when the rat misplaced its paw and the limb fell between the rungs. The total number of foot-faults was quantified.

### Statistical analysis

Data are expressed as means ± standard error of the mean (SEM). The data were analyzed using Student's t-test for the open-field, elevated plus-maze and foot-fault tasks. Locomotor and exploratory parameters were analyzed across time using two-way ANOVA followed by the Bonferroni post-hoc test. A *p* value *<*0.05 was considered significant for all parameters. Data analysis was performed in Microsoft Excel 2007 and GraphPad Prism 5.0 softwares.

## Results

### Experimental cirrhosis

BDL rats exhibited yellowish fur and tails and enlarged abdomens. The body weights were not different between the control (225.5±5.0 g) and BDL (226.4±5.4 g) rats at the sixth week postsurgery. All BDL rats had ascites and their livers were tinged yellow with cystic bile duct remnants. Hepatomegaly and splenomegaly were observed in all BDL rats. One BDL rat in each group died prior to sample collection.

### Histopathological and biochemical characterization

Histological analysis of the H&E- and picrosirius-stained liver tissue samples revealed that all BDL rats exhibited bile duct proliferation, disturbed cytoarchitecture, formation of septa between portal areas and a noticeable increase in collagen fibers. Control rats exhibited normal liver parenchyma. The spleen tissue samples of the BDL rats exhibited blood congestion, which is in agreement with portal hypertension. Serum AST, alkaline phosphatases and bilirubin (total and direct) were significantly increased in the BDL rats (Table 1). Albumin concentration was lower in the BDL rats compared to the control rats. These findings confirmed chronic cholestatic liver cirrhosis in the BDL rats.

### Behavioral studies and Open-field task

**Activity.** BDL rats exhibited a significantly smaller total distance traveled in the open arena (*t* = 2.788, *df* = 9, *p* = 0.0211), a higher duration of immobile episodes (*t* = 3.0801, *df* = 9, *p* = 0.0042) and consequently, a smaller duration of mobile episodes, compared to the control rats (Figs. 1A,B). When analyzing the distance traveled across time it was observed that both groups presented the same pattern of behavior, i.e. both the control and BDL rats traveled larger distances during the first minutes and then decreased their locomotor activity over time. However, the BDL group exhibited a significantly smaller distance traveled in the first 6 minutes compared to the control rats (Fig. 2A, *F* = 15.49, *df = *14, *p*<0.0001 for variable time). The same pattern of behavior was observed in mobility across exploration time (Fig. 2B, *F* = 19.46, *df* = 14, *p*<0.0001 for variable time). The total number of stops during the exploratory activity was not different between the BDL and control rats (Fig. 1C), albeit, the distance traveled between these stops was significantly smaller in the BDL group (Fig. 1D, *t* = 2.919, *df* = 9, *p* = 0.0171). The total number of rearings during this task was significantly decreased in the BDL group (Fig. 1E, *t* = 3.230, *df* = 9, *p* = 0.0103), and the analysis across time revealed that this difference was only apparent during the first two minutes of the task (Fig. 2C, *F* = 10.73, *df* = 14, *p*<0.0001 for variable time). Grooming behavior did not differ between groups (Fig. 1F).

#### Temporal organization of behavior

A significant increase was observed in the time that the BDL animals remained in the home-base area compared to the control rats (Fig. 3A, *t* = 2.348, *df* = 9, *p* = 0.0434). The number of trips during this task was not different between the groups (Fig. 3B), but the length of each trip was considerably shorter in the BDL group (Fig. 3C, *t* = 3.983, *df* = 9, *p* = 0.0032). The number of stops made in trips between two successive stops at the home-base (stops/trips) was not different between groups (Fig. 3D).

#### Spatial distribution of activity

The percentage of the distance traveled within the center zone was not different between the BDL and control rats (Fig. 4A). However, the percentage of time spent in the center of the arena was significantly smaller in the BDL rats compared to the control rats (Fig. 4B, *t* = 2.824, *df* = 9, *p* = 0.0199).

### Elevated plus-maze

No difference in the percentage of the number of entries either in the percentage of time spent in the closed arms was observed between the BDL and control rats (Table 2).

### Foot-fault

No difference in the total number of foot-faults was observed between the BDL and control rats (Table 2).

## Discussion

The BDL rat is an experimental model of secondary biliary cirrhosis that leads to chronic HE type C [21]. Studies described that rats with chronic liver failure developed by different models, exhibit impaired locomotor and exploratory behaviors [9], [12], [14]. In the present study the BDL rats, which exhibited chronic liver disease, had impaired locomotor and exploratory activities in the open-field task, characterized by a decrease in the total distance traveled and an increased immobility time. Furthermore, the BDL rats also traveled shorter distances between stops during the exploratory activity, but the total number of stops was not different from the control rats. Reinforcing the concept of altered locomotor and exploratory activities, the total number of rearings was smaller in the BDL rats. These results are in accordance with previous studies that demonstrated that different models of chronic liver disease exhibit locomotor dysfunction. Specifically, rats subjected to PCS have been shown to exhibit hypolocomotion in the open-field task [12], [14], [24] and BDL rats have been shown to exhibit a decreased locomotor activity when underwent the open-field behavioral task [8], [9]. Contrary to these results, Méndez et al. [28] did not observe differences in locomotor behavior when studying the PCS rats and the thioacetamide-induced liver failure rat models. However, the majority of the locomotor and exploratory behavior studies have just focused only on parameters that depict animal activity in the apparatus. The present study explored the open-field task in more detail, since the temporal and spatial structures of the behaviors were also investigated.

It is known that rats after being introduced to the open field establish a home base, which is the most visited location in the arena in which they spend an extended period of time and this is the place from which the rats will initiate trips [20]. Further analysis of the temporal (sequential) structure of the locomotor behavior of the BDL rats demonstrated that these animals remained in the home base area for longer periods of time than the control rats, but both groups performed the same number of exploratory trips. Therefore, the decreased in the total distance traveled by the BDL rats in the open-field is likely not related to the number of trips nor to the number of stops per trip performed by the animals. However, the distance traveled per trip was smaller in the BDL group, which consequently reduced the total distance traveled. With regard to the spatial distribution of locomotion, the BDL rats did not differ from the control rats in the percentage of distance traveled in the center zone of the arena. However, the time spent in this area was decreased in the BDL rats. This pattern of behavior could be interpreted as avoidance of the open area, which suggests an anxiety-like behavior. For this reason, the elevated plus-maze task was conducted to investigate anxiety; interestingly, it was found that the BDL rats did not exhibit greater anxiety behavior than the control rats. In agreement with this result, Sergeeva et al. [13] did not observe anxiety behavior in PCS rats, however, the animals moved less and more slowly in the open-field task compared to the control rats. In addition, it has been shown that rats with chronic liver failure induced by thioacetamide intoxication did not show anxiety behavior; it is notable, however, that they did not display locomotor and exploratory deficits either [28]. Furthermore, the diminished locomotor activity exhibited by the BDL rats in the present study was not a consequence of impaired motor coordination because the BDL rats were as coordinated as the control rats when performing the foot-fault task. In accordance with this result, Jover et al. [9] demonstrated that BDL rats exhibited mild coordination impairment in the the rotarod test whereas there was no statistical difference between the BDL and control rats in the beam walking test. Taken together, our results clearly demonstrate altered spatio-temporal organization of locomotor and exploratory activities in the BDL rats, but the hypolocomotion observed did not appear to be the result of anxiety or impairment of motor coordination (schematic representation in Fig. 5). It should be noted that we found no differences between the locomotor and exploratory behaviors of female and male BDL rats (data not shown).

Patients with HE exhibit impaired motor function and coordination and present the clinical manifestations such as asterixis, ataxia, dysarthria, rigor, tremor, hypomimia and hypokinesia [3], [4], [29]. It has been described that disturbances in motor function, such as hypokinesia and bradykinesia, are a consequence of altered basal ganglia function [6], [29]. Amondio et al. [30], suggested that motor alterations were due to altered neuronal circuits between the basal ganglia and prefrontal cortex. The mechanisms underlying the HE-induced alterations in these cerebral areas are still unclear, although several studies have been undertaken to elucidate these mechanisms. Joebges et al. [5] showed in patients with minimal HE, i.e. a condition in patients with chronic liver disease and signs of HE that are not clinically manifested, that cortical brain areas such as the cingulate gyrus as well as the frontomesimal and parietal cortex, which are related to movement initiation, exhibited decreased glucose uptake.

Studies in animals have further investigated the underlying mechanisms of induced motor alterations during HE. Oria et al. [31] found that PCS and BDL rats did not exhibit alterations in motor-evoked potentials in conscious rats and suggested that the behavioral abnormalities that have been described might be a consequence of the dysfunction of other neuronal circuits. Another study using PCS rats demonstrated an increase in extracellular glutamate and metabotropic glutamate receptor mGluR1 activation in the substantia nigra reticulate, which may be related to the reduced motor activity observed in these animals [12]. It has been shown that the function of the neuronal pathway related to motor function, i.e. the circuits between the basal ganglia, thalamus and cortex was impaired in PCS rats [11]. In addition, increased levels of neurosteroids and consequently dysfunction in GABAergic neurotransmission might also participate in the altered locomotor activity observed in PCS rats [14]. In this context, it may be important to note that BDL rats have been shown to have impairments of the biosynthesis of neurotransmitter GABA [23].

Overall, different studies have described alteration in the function and neurochemistry of brain areas that are related to motor function, which have been related to the dysfunctions in the locomotor and exploratory activities. However, it is important to consider that other brain areas might be involved. According to that, it has been demonstrated that different rat models of chronic liver disease and HE type B and C exhibit deficits in tasks related to visual orientation [28]. Moreover, it has been described that patients with HE have impairments in visual perception, visual orientation and attention [32]–[34]. BDL rats, in the present study, exhibited an altered spatio-temporal organization of locomotor and exploratory behaviors. It should be considered that visual and spatial perception deficits might also interfere with locomotor and exploratory activities.

In conclusion, BDL rats with chronic liver disease and mild HE exhibited altered spatio-temporal organization of locomotor and exploratory activities compared to control rats. The mechanisms underlying these behavioral impairments are not completely understood, and further studies will be fundamental for the elucidation of which brain areas and neurochemical mechanisms that are involved.
